# Quantitative Assessment of Acetabular Defects in Revision Hip Arthroplasty Based on 3D Modeling: The Area Increase Ratio (*AIR*) Method

**DOI:** 10.3390/bioengineering11040341

**Published:** 2024-03-30

**Authors:** Giuseppe Marongiu, Antonio Campacci, Antonio Capone

**Affiliations:** 1 Orthopaedic Clinic, Department of Surgical Sciences, University of Cagliari, 09124 Cagliari, Italy; profantoniocapone@gmail.com; 2 IRCCS Ospedale Sacro Cuore Don Calabria, Negrar, 37024 Verona, Italy; antonio.campacci@gmail.com

**Keywords:** revision hip arthroplasty, acetabular bone defect, classification, 3D modeling, 3D printing

## Abstract

The most common classifications for acetabular bone defects are based on radiographic two-dimensional imaging, with low reliability and reproducibility. With the rise of modern processing techniques based on 3D modelling, methodologies for the volumetric quantification of acetabular bone loss are available. Our study aims to describe a new methodology for the quantitative assessment of acetabular defects based on 3D modelling, focused on surface analysis of the integrity of the main anatomical structures of the acetabulum represented by four corresponding sectors (posterior, superior, anterior, and medial). The defect entity is measured as the area increase ratio (AIR) detected in all the sectors analyzed on three planes of view (frontal, sagittal, and axial) compared to healthy hemipelvises. The analysis was performed on 3D models from the CT-scan of six exemplary specimens with a unilateral pathological hemipelvis. The AIR between the native and the pathological hemipelvis was calculated for each sector, for a total of 48 analyses (range, +0.93–+171.35%). An AIR of >50% were found in 22/48 (45.8%) sectors and affected mostly the posterior, medial, and superior sectors (20/22, 90.9%). Qualitative analysis showed consistency between the data and the morphological features of the defects. Further studies with larger samples are needed to validate the methodology and potentially develop a new classification scheme.

## 1. Introduction

The number of revision total hip arthroplasty (rTHAs) procedures is predicted to dramatically increase in a short time, with rates over 50% [1,2]. Additionally, the risk of a second revision procedure after rTHA reaches 19% according to recent studies [3,4]. The main cause for THA failure is aseptic loosening of the implant on the acetabular side, which is often associated with severe bone defects [5,6]. The accurate assessment of the bone loss and remaining bone stock is essential for planning the revision surgery procedure and choosing the appropriate implant design; therefore, classifying acetabular defects remain a major issue. In current medical practice, classification schemes of acetabular bone defects mostly rely on traditional radiographs, which can provide a solely two-dimensional image of more complex anatomy [7,8,9,10,11,12]. One of the most common, the Paprosky classification, is based on the analysis of the implant migration and the remaining radiological anatomical landmarks, and provides different indications for surgical reconstruction according to the severity and localization of bone defects [7]. However, different authors reported low reliability and reproducibility due to a lack of accuracy in standard radiograph analysis and the need for subjective evaluation of bone defect features [13,14,15]

CT (computed tomography) scans offer a more accurate three-dimensional view, showing anatomical structures that otherwise would be covered by the overlap of the metal implant in plain radiographs [16]. With the rise of modern 3D-modelling software, CT images can be elaborated and used to recreate tridimensional anatomical replicas of the patients’ anatomy [17,18,19].

In this scenario of advanced imaging techniques, different studies proposed new methodologies for the assessment of acetabular bone defects based on 3D models. Zhang et al. proposed a new system that relies on a qualitative analysis of acetabular bone defects on 3D models of the pelvis without a quantitative assessment, focusing on the integrity of the supporting structures to achieve sufficient implant fixation [20]. The system needed a subjective estimation of the bone loss, and they reported excellent reliability and reproducibility when compared to X-rays. Recently, Meynen et al. used 3D models to analyze the accuracy of the Acetabular Defect Classification (ADC), a qualitative classification originally elaborated by Wirtz et al. [21,22]. On 3D-CT imaging with statistical shape models, bone loss volume was quantified, and this analytical defect information was used by the raters to classify defects, resulting in doubling the intra- and inter-rater reliability and in upscaling the acetabular defect classification when compared to standard radiographs [23].

The first attempts for a reliable and precise methodology for computerized volumetric quantification of acetabular bone loss was the total radial acetabular bone loss (TrABL) method by Gelaude et al., who developed advanced CT-based image processing for 3D anatomical reconstruction [24]. The output of the analysis consisted of a ratio and a graphical spatial representation of the bone defect in the lateral view of the acetabulum. The methodology offered precise information in terms of volume of bone loss and spatial localization around the acetabulum but did not provide indications about the residual structural support of the remaining bone stock. Therefore, the authors suggested its use as a support for complex revision case and preexisting classification systems [24].

Similarly, Hettich et al. validated an analytic method of acetabular bone loss pointing out the opportunity of quantifying the volume of acetabular bone defects using statistical shape models and dividing the acetabulum into four different anatomical sectors according to the main structural areas of the acetabulum (anterior wall and column, the posterior wall and column, the superior dome, and the medial wall) [25]. Later on in another study, the authors applied the methodology on 50 cases, where the bone loss was expressed as a percentage of decreased bone volume on the different sectors of the acetabulum, and added a qualitative analysis that described the morphology of the most common defects [26].

The methodologies previously described focused on the analysis of the bone defects in terms of bone volume loss. This quantitative assessment, although objective and reliable, do not offer an overall intuitive comprehension of the extent and severity of the defect. Moreover, providing only a visual output of the lateral view of the acetabulum makes it difficult to understand the morphology and extension of the defect and the integrity of the main structural areas. This could potentially limit the use of these methodologies in the daily clinical practice when the surgeon is called to identify the implant design according to residual supportive bone surfaces and consequently its fixation points.

In this study, we aim to describe a method of quantitative assessment of acetabular defects based on 3D CT-scan models that allow both an objective quantification and a visual output of the defect in the three different planes: the axial, sagittal, and coronal planes. The methodology relies on the analysis of the residual bone stock of each main structural area of the acetabulum expressed as *sectors*, quantifying the ratio of bone surface loss compared to the healthy hemipelvis.

## 2. Material and Methods

The methodology was retroactively applied on CT scan specimens of six patients selected from the data set of our institution. We included specimens of patients who underwent THA revision surgery for aseptic loosening and were diagnosed with acetabular bone defects, with a contralateral healthy native hemipelvis. The CT scans were visually analyzed, and the acetabular defects were classified by two senior surgeons (AC and GM) according to the Paprosky classification [7] (Table 1).

The provision of the CT scans was approved by the teaching hospital ethics committee (Prot. PG/2021/9935).

**Figure 1:**
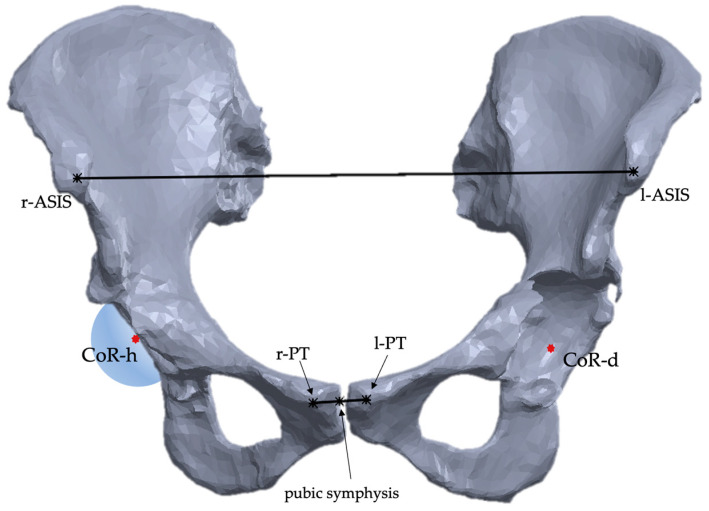
Landmarks used for the mirroring procedure of the healthy hip center of rotation (CoR-h) on the defected side (CoR-d). This case shows an acetabular defect of the left hemipelvis with a healthy right hemipelvis. The right and left anterior superior iliac spine (r-ASIS, l-ASIS) and the right and left pubic tubercle (r-PT, l-PT) are identified as reference landmarks.

The methodology consisted of three phases: (1) *pre-processing and mirroring*, (2) *views and sector definition*, and (3) *analysis* (Figure 1). The pre-processing included CT-scan acquisition, segmentation of bone structures and metal implants, and the mirroring of the healthy hemipelvis on a solid model of the pathological hemipelvis. In the *views and sector definition* phase, three different planes were identified corresponding to a frontal view, a sagittal section view, and an axial section view of the acetabulum. Four different sectors corresponding to the main structural acetabular areas were identified on the native pelvis and pathological pelvis. In the *analysis* phase, measurements of bone loss in terms of areas of bone stock lost compared to the native acetabulum were made.

### 2.1. Pre-Processing and Mirroring

CT scans of the specimens included in the study were performed with a 3 mm slice thickness and a pixel size of 0.80 mm. During the acquisition, a preliminary metal artifacts protocol was applied using MAR software (version 2013, General Electric Healthcare, Chicago, IL, USA).

Digital imaging and communications in medicine (DICOM) files of CT scans of the pelvises were imported into Mimics Innovation Suite 3D modelling software (version 23.0, Materialise, Leuven, Belgium). A single trained operator, GM, performed further steps under clinical supervision of a senior surgeon, AC. A *reduce scattering* protocol was applied for residual metal artifact noise. Bony parts and metal implants were manually segmented, obtaining three-dimensional separate objects of the two hemipelvises. Thresholding was manually edited to preserve both cortical and cancellous bone. After post-processing with filling techniques, a smooth factor of 0.70 mm, and a wrap factor of 1.0 mm, the 3D objects were exported as standard triangulation language (STL) mesh and processed in 3-Matic software (version 15.0, Materialise, Leuven, Belgium).

After mesh correction, a sphere was fitted lying on the acetabulum surface to identify the center of rotation of the native hip. Considering that the disrupted anatomy of the pathological hemipelvis could not allow us to precisely identify the hip center of rotation, using the *mirroring tool*, the native hemipelvis was mirrored on the pathological side, which was the native hip center of rotation (CoR) and represents a pivoting reference landmark for the classification system (Figure 1).

### 2.2. Planes and Sectors Definition

In this phase, the acetabulum anatomical supporting structures, the posterior and anterior column, the superior dome, and medial wall were identified as 4 different sectors according to their clinical relevancy.

Starting on the healthy hemipelvis, the sectors were defined on 3 different planes: *a frontal plane, a sagittal plane, and an axial plane*, orthogonal to each other.

**Figure 2:**
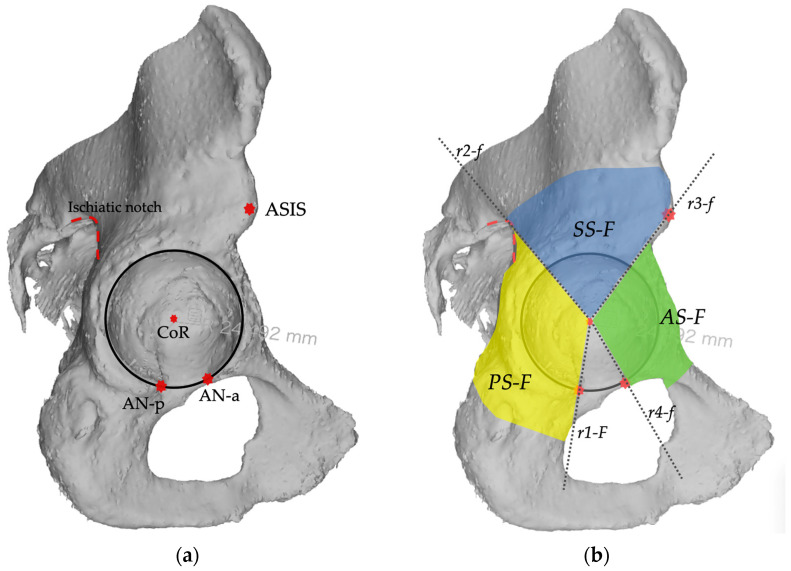
Landmarks and sectors construction on the *frontal plane.* (**a**) In the frontal plane view, the landmarks for sectors description are identified: the acetabular rim (black circle), the center of rotation (CoR), the anterior superior iliac spine, the ischiatic notch (red dotted line), and the anterior and posterior aspect of acetabular notch (AN-p, AN-a); (**b**) In the frontal view, four reference lines (r1-F, r2-F, r3-F, r4-F) are used to describe three sectors: the posterior (PS-F), the superior (SS-F), and the anterior (AS-F).

The *frontal plane* corresponded to the lateral anatomical view of the acetabulum and was defined by the acetabular rim border. In this plane, 3 sectors are identified, the posterior column, the superior dome, and the anterior column. The *posterior sector* (*PS-F*) is defined by a line (*r1-F*) drawn caudally from the center of rotation to the projection of anterior aspect of the acetabular notch, by a line (*r2-f*) drawn cranially from the center of rotation (CoR) towards the projection of the ischiatic notch. The *anterior sector* (*AS-F*) is defined by a line (*r3-F*) drawn cranially from the center of rotation towards the projection of the anterior superior iliac spine (ASIS), and a line (*r4-F*) drawn caudally from the center of rotation towards the projection of the posterior aspect of the acetabular notch. The *superior sector* (*SS-F*) is defined by line *r2-F* and line *r3-F* (Figure 2).

**Figure 3:**
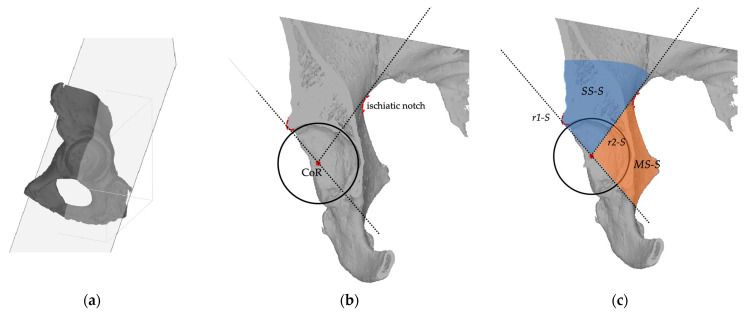
(**a**) the *sagittal plane* crosses the acetabulum at the 18 o’clock and 12 o’clock position. (**b**) In the sagittal plane view, the landmarks for the sector description are the acetabular rim (black circle), the center of rotation (CoR), the ischiatic notch (red dotted line), and the superior anterior margin of acetabular rim (red dotted line); (**c**) In the axial view, two reference lines (*r*1-*S*, *r*2-*S*) are used to describe two sectors: the superior (SS-S), and the medial (MS-S).

The *sagittal plane* is orthogonal to the frontal plane and lies on a straight line connecting the CoR and the 18 o’clock and 12 o’clock position around the acetabular rim as reference landmarks and crosses the medial wall and the acetabular superior dome. In this plane, 2 sectors are identified. The *superior sector* (*SS-S*) is defined by a line (*r1-S*) drawn from the center of rotation to the projection of the superior aspect of the acetabular rim, and by a line (*r2-S*) drawn posteriorly from the center of rotation (CoR) towards the projection of the ischiatic notch. The *medial sector* (*MS-S*) is defined by the *r2-S* line and the caudal extension of the *r1-S* line (Figure 3).

**Figure 4:**
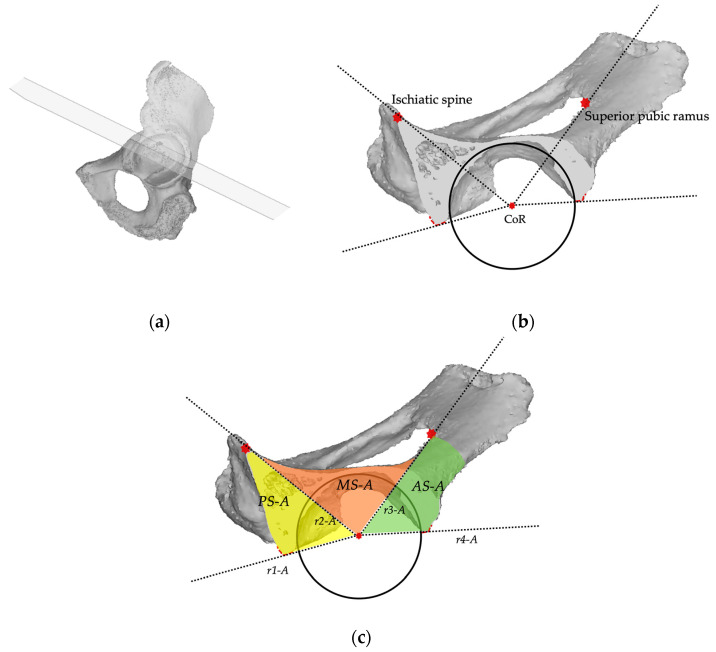
(**a**) the *axial plane* crosses the acetabulum at the 9 o’clock and 15 o’clock position. (**b**) In the sagittal plane view, the landmarks for sectors description are the acetabular rim (black circle), the center of rotation (CoR), the ischiatic spine, and the anterior and posterior margin of acetabular rim (red dotted lines), and the posterior part of the superior pubic ramus; (**c**) In the axial view, four reference lines (r1-A, r2-A, r3-A, r4-A) are used to describe three sectors: the posterior (PS-A), the medial (MS-A), and the anterior (AS-A).

The *axial plane* is orthogonal to the sagittal plane and lies on a straight line connecting the CoR and the 9 o’clock and 15 o’clock position around the acetabular rim, and crosses the posterior column, the medial wall, and anterior column. In this plane, three sectors are identified. The *posterior sector* (*PS-A*) is defined by the line (*r1-A*) drawn posteriorly from the center of rotation to the posterior acetabular rim, and the other line (*r2-A*) drawn from the center of rotation (CoR) towards the projection of the posterior part of the superior pubic ramus connecting to the obturator foramen. The *anterior sector* (*AS-A*) is defined between the line (*r3-A*) drawn from the center of rotation towards the projection of the ischiatic spine (IS) and the line (*r4-A*) drawn from the center of rotation to the anterior acetabular rim. The *medial sector* (*MS-A*) is defined by line *r2-A* and line *r3-A* (Figure 4).

**Figure 5:**
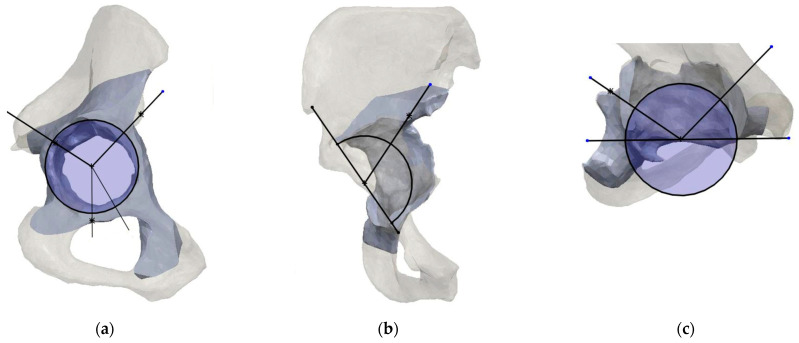
Landmarks and sectors definition on the pathological hemipelvis of specimen 1. The native center of rotation, acetabular rim, and reference lines are overlapped on the pathological acetabulum on the three planes; (**a**) frontal, (**b**) sagittal, and (**c**) axial.

On the pathological side, the procedure is repeated using the mirrored native hip center of rotation as a pivotal reference point, as well as the remaining anatomical landmarks, as shown in Figure 5.

### 2.3. Analysis

**Figure 6:**
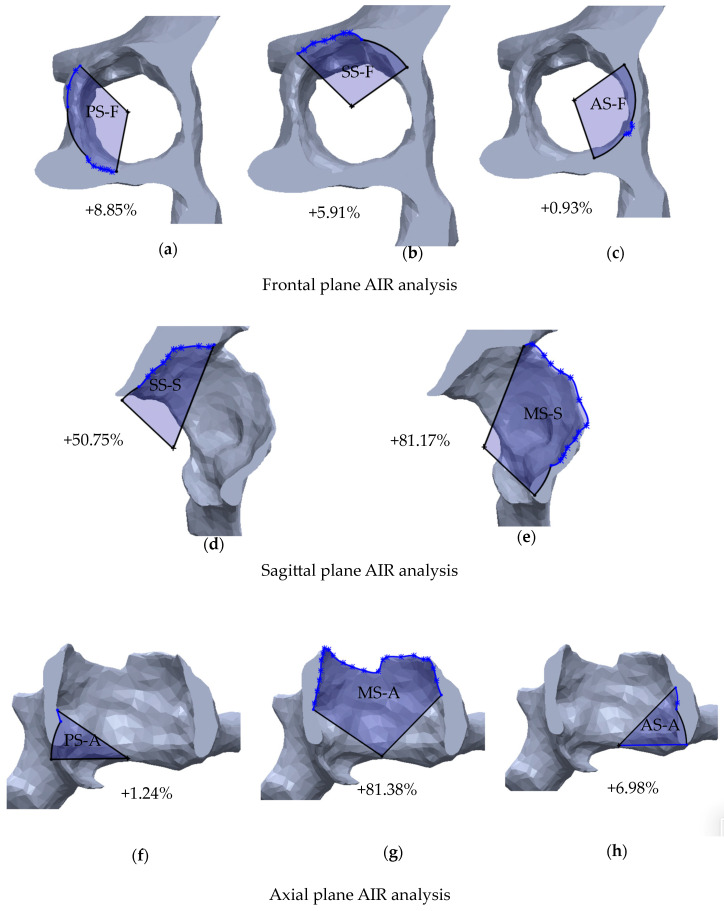
Area increase ratio (AIR) analysis performed on specimen one. (**a**–**c**) In the frontal plane, AIR values (%) are calculated for the posterior (PS-F), superior (SS-F), and anterior (AS-F) sectors; (**d**,**e**) In the sagittal plane, AIR values are calculated for the superior (SS-S) and medial (MS-S) sectors; (**f**–**h**) In the axial plane, AIR values are calculated for the posterior (PS-A), medial (MS-A), and anterior sectors (AS-A).

In this phase, the different sectors are transformed into polygons using the acetabular rim as the third side of the polygon. The same procedure is repeated on the pathological side using the furthest borders of the bone defect as the third side. The areas of the sectors are then measured (mm^2^), and the bone defect entity is expressed as the percentual increase of the defected sector area (*A_def_*) compared to the corresponding healthy sector (*A_nat_*), using this equation (Figure 6):

$$\mathrm{Area}\mathrm{Increase}\mathrm{Ratio}\;(\mathrm{AIR})\;\left(1-\frac{Adef}{Anat}\right)\times 100\%$$

Conversely, the percentual increase of the area represents the amount of bone loss that occurred in a sector. Defects are then categorized into minimal, moderate, severe, or massive according to the area increase ratio (*AIR*) values detected in each sector (Table 2). The grading in minimal, moderate, severe, and massive was inspired by clinical consideration derived by most used classification systems [7,8,10] and the thresholds as similarly proposed by Gelaude and Hettlich [24,25].

## 3. Results

### 3.1. Quantitative Defect Assessment

The suggested method was applied on six exemplary specimens (Table 3).

**Figure 7:**
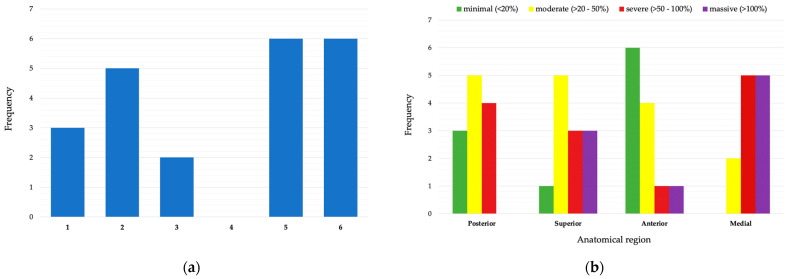
(**a**) Histogram of AIR ratios exceeding 50% (severe), per specimen (specimens named one to six, as specified in Table 2). (**b**) Histogram of AIR ratios according to the grading per anatomical region (Posterior: posterior sectors, Superior: superior sectors, Anterior: anterior sectors, Medial: medial sectors).

Acetabular rim diameters of the native hemipelvis ranged from 52 to 66 mm. The area increase ratio (AIR) between the native hemi-pelvis and the pathological hemi-pelvis was calculated for each sector, for a total of 48 single analyses. Minimal defects, ranging from 0 to +17.75% of the AIR, were found in 11/48 (22.91%) sectors analyzed, while a moderate defect (range, +28.61–+49.20%, AIR) was detected in 15/48 (31.25%) sectors. Severe defects (range, +109.02–+91.30%, AIR) were found in 13/48 (27.08%) sectors analyzed, while massive defects (range, +50.75–+171.35%, AIR) were identified only in 9/48 (18.75%) sectors. Minimal defects were mostly localized in the anterior sectors (6/11, 54.54%). Most severe defects affected the posterior, superior, and medial sectors (12/13, 92.30%). Massive defects were most likely located in superior and medial sectors (8/9, 88.88%). Only in one case did a massive defect involve an anterior sector (Figure 7).

### 3.2. Qualitative Defect Assessment

Specimens one and three showed a IIC Paprosky type that describes an acetabular defect with a distorted rim but intact supportive columns, and an all-medial migration of the implant. Similarly, the AIR analysis showed minimal defects for anterior and posterior sectors, and severe to massive defects in the medial sector. Additionally, in specimen one, the sagittal plane showed a severe defect in the superior sector which was not visible in the frontal analysis. This highlights that the analysis on two planes for each sector is crucial to better understanding the defect’s morphology, extension, and severity. Specimens two and six showed a IIIB Paprosky type defect, which is characterized by superior migration of the implant and severe posterior and medial osteolysis, with bone loss spanning from the 9 o’clock to 5 o’clock position. AIR analysis showed, as well, massive defects located in the superior and medial sectors and moderate to severe defects in the posterior sectors. In Specimen 4, an IIA Paprosky type, were found as expected, with only minimal and moderate AIR defects. Specimen 5 was classified as an IIIA Paprosky type, which implies a severe superior migration of the implant and severe ischial and medial defects, with bone loss at the 10 o’clock to 2 o’clock position.

## 4. Discussion

The Paprosky classification represents the most common and recognized scheme, first offering a practical surgical algorithm that relies on the qualitative assessment of the integrity of the main supportive anatomical structure of the acetabulum [7]. As for the other most used acetabular defect classification systems, they relies on 2D radiographs, with poor results in terms of reliability and accuracy between preoperative planning and intraoperative findings [13,14,15]. The development of modern diagnostic and tridimensional reconstruction techniques inspired different authors to use 3D models to anticipate the intraoperative reality and consequently develop new quantitative and reproducible classification methods [19,20,23,26]. Significant efforts were made to switch from a qualitative evaluation to a quantitative estimation of the bone defects. First, Gelaude et al. validated a methodology for the quantification of acetabular defects that relies on the volumetric analysis of bone loss and provides a schematic visual output of the defected acetabulum [24]. Then, Hettlich et al. proposed a similar volumetric analysis, adding an anatomic visual output of the lateral view of the acetabulum and identifying different anatomical sectors. They highlighted the need for spatial information aside from the volumetric value of bone loss to assess the integrity of the main supporting structure of the acetabulum [25]. Conversely, the sole visualization in the lateral view of the acetabulum does not offer an overall display of the morphology and extension of the defect, which most likely have erratic shapes, widths, and depths. Nevertheless, these methodologies showed the potential of 3D models analysis to improve the existing classification schemes, as reported by Meynen et al. [23].

Starting from this premise, we elaborated a methodology aiming to provide a quantitative assessment of acetabular defects associated with a visualization of the structural anatomical area of the acetabulum, in the three planes of the space. The methodology is based on the quantitative analysis of bone defects, expressed as the area of bone loss detected in the four main anatomical sectors of the acetabulum, first measured on a plane corresponding to the lateral anatomic view of the acetabulum (the *frontal plane*) and then two additional planes crossing the acetabulum (the *sagittal* and *axial plane*). In the *frontal plane,* we analyzed the integrity of the acetabular rim and surrounding structures (posterior column, superior dome, and anterior column). The *sagittal plane* was meant to offer a comprehensive cross view of the superior dome and the supero-lateral or medial extension of the defect and integrity of the medial wall. The *axial plane* crosses the acetabulum at its equatorial level and shows the integrity of the mid part of the posterior and anterior column, as well as the medial wall. The reference landmarks we used to determine the sectors were manually detected, and easily reproducible in both sides of all the specimens, allowing us to border the sectors even when severe bone loss occurred.

We believe that the comparison of defected sector’s area to the healthy corresponding area using the raw square millimeter measure could be not efficient in representing the impact of a determined defect among acetabula of different dimensions. Therefore, the quantitative value was expressed as a ratio of the area increase (AIR), and then categorized into minimal, moderate, severe, or massive to describe and scale different defect types according to patient-specific anatomy. The grading system was inspired by the most used classification systems, with the aim to offer an intuitive and feasible tool for clinical application [7,8,9,10,12].

In our preliminary analysis, we found a majority of moderate and severe defected sectors (28/48, 58.3%) in the posterior and superior sectors. Minimal defects were found specially in anterior sectors (6/11, 54.5%), while massive defects mostly involved superior and medial sectors (8/9, 88.8%). This prevalence of moderate–severe defects was consistent with the severity of Paprosky types detected, all II and III types. Moreover, there was a visual correlation between the morphology of Paprosky types and AIR defects. Accordingly, AIR severe defects were found in the posterior, superior, and medial sectors. Interestingly, completely different AIR gradings can coexist in the same hemipelvis; a sector can be massively affected by bone loss while another sector can be completely intact, suggesting that the methodology seems able to comprehensively describe the features of an acetabulum defect.

The proposed methodology has several limitations. First, we described the methodology only on specimens with a contralateral healthy hemipelvis, using “a mirroring technique”. In the future, the procedure will be applied to specimens with both defected hemipelvises with the support of statistical shape models (SSMs) as described by other authors [21,25]. The second limitation is the manual processing of the 3D models for metal artefacts reduction, segmentation, and landmarks identification. To be clinically feasible and to improve the accuracy of the method, some of these steps should be automated with the support of artificial intelligence (AI). Third, the small number of specimens did not allow us to validate the methodology, so further studies with larger samples are needed.

We believe that the main novelty and strength of our methodology is the change of focus from measuring the volume of bone loss to measuring the area of bone loss. Through the area increase ratio (AIR), we described the defect as a “missing supporting surface”, offering a visual perception of morphology, location, and extent of the defect on three different spatial planes.

Nevertheless, the methodology needs to be validated in terms of accuracy and reliability. Further analysis could enhance the definition of defect categories and the grading system, especially for massive defects, as in the case of multiple revision procedures or in post-traumatic defect and oncologic surgery settings. Using SSMs technology, the methodology could be merged into 3D CT-based planning software for preoperative surgical decision making and support the development of new treatment algorithms, including patient-specific surgical strategies.

## Figures and Tables

**Table 1:** Specimens characteristics and defect type according to Paprosky classification.

Spec	Age	Sex	Side	Diagnosis	Paprosky Type
1	67	F	right	Aseptic loosening	IIC
2	78	F	right	Aseptic loosening	IIIB
3	84	M	left	Aseptic loosening	IIC
4	78	F	left	Aseptic loosening	IIA
5	74	M	right	Aseptic loosening	IIIA
6	69	M	right	Aseptic loosening	IIIB

**Table 2:** Based on the area increase ratio (AIR), a defected sector is graded into four progressive categories according to the severity of the bone loss detected.

Minimal	Moderate	Severe	Massive
0–20%	>20–50%	>50–100%	>100%

**Table 3:** Surface values of the defected sectors (area, mm^2^) and area increase ratios (AIR) of the six specimens.

		Frontal Plane			Sagittal Plane		Axial Plane		
Spec.	Acetabular Diameter	PS-F	SS-F	AS-F	SS-S	MS-S	PS-A	MS-A	AS-A
1	66 mm	1239.78 mm^2^	1038.92 mm^2^	1020.5 mm^2^	976.34 mm^2^	1989.53 mm^2^	338.73 mm^2^	1723.69 mm^2^	457.5 mm^2^
		+5.91%	+8.85%	+0.93%	+50.75%	+87.17%	+1.84%	+81.38%	+6.98%
2	56 mm	1194.42 mm^2^	1033.49 mm^2^	1046.81 mm^2^	1196.34 mm^2^	1443.84 mm^2^	369.56 mm^2^	1569.34 mm^2^	572.02 mm^2^
		+47.42%	+35.43%	+53.11%	+171.35%	+82.62%	+28.61%	+136,47%	+109.02%
3	56 mm	963.31 mm^2^	892.65 mm^2^	1020.97 mm^2^	494.59 mm^2^	870.91 mm^2^	320.26 mm^2^	696.71 mm^2^	273.66 mm^2^
		+36.53%	+30.93%	+17.75%	+5.15%	+114.42%	+4.02%	+117.19%	+0.00%
4	52 mm	1039.78 mm^2^	868.92 mm^2^	680.5 mm^2^	580.34 mm^2^	989.53 mm^2^	300.73 mm^2^	829.69 mm^2^	307.5 mm^2^
		+43.09%	+46.66%	+8.43%	+44.35%	+49.97%	+45.65%	+40.64%	+15.83%
5	54 mm	1189.42 mm^2^	1220.49 mm^2^	867.81 mm^2^	706.34 mm^2^	1143.84 mm^2^	469.56 mm^2^	978.34 mm^2^	332.02 mm^2^
		+57.88%	+72.00%	+36.51%	+72.30%	+55.59%	+75.74%	+58.4%	+30.48%
6	58 mm	1363.31 mm^2^	1642.65 mm^2^	1220.97 mm^2^	1194.59 mm^2^	1830.91 mm^2^	640.26 mm^2^	1596.71 mm^2^	433.66 mm^2^
		+80.12%	+124.61%	+31.28%	+136.76%	+124.25%	+93.87%	+129.01%	+47.72%

## Data Availability

The raw data supporting the conclusions of this article will be made available by the authors on request.
